# Genome content analysis yields new insights into the relationship between the human malaria parasite *Plasmodium falciparum* and its anopheline vectors

**DOI:** 10.1186/s12864-017-3590-0

**Published:** 2017-02-27

**Authors:** Sara J. Oppenheim, Jeffrey A. Rosenfeld, Rob DeSalle

**Affiliations:** 1 Sackler Institute for Comparative Genomics, American Museum of Natural History, New York, NY 10024 USA; 2 Cancer Institute of New Jersey, Rutgers University, New Brunswick, NJ USA

**Keywords:** Gene ontology, Genome content, Automatic annotation, Malaria, Plasmodium falciparum, Anopheles, Vector-parasite interactions

## Abstract

**Background:**

The persistent and growing gap between the availability of sequenced genomes and the ability to assign functions to sequenced genes led us to explore ways to maximize the information content of automated annotation for studies of anopheline mosquitos. Specifically, we use genome content analysis of a large number of previously sequenced anopheline mosquitos to follow the loss and gain of protein families over the evolutionary history of this group.

The importance of this endeavor lies in the potential for comparative genomic studies between *Anopheles* and closely related non-vector species to reveal ancestral genome content dynamics involved in vector competence. In addition, comparisons within *Anopheles* could identify genome content changes responsible for variation in the vectorial capacity of this family of important parasite vectors.

**Results:**

The competence and capacity of *P. falciparum* vectors do not appear to be phylogenetically constrained within the Anophelinae. Instead, using ancestral reconstruction methods, we suggest that a previously unexamined component of vector biology, anopheline nucleotide metabolism, may contribute to the unique status of anophelines as *P. falciparum* vectors. While the fitness effects of nucleotide co-option by *P. falciparum* parasites on their anopheline hosts are not yet known, our results suggest that anopheline genome content may be responding to selection pressure from *P. falciparum*. Whether this response is defensive, in an attempt to redress improper nucleotide balance resulting from *P. falciparum* infection, or perhaps symbiotic, resulting from an as-yet-unknown mutualism between anophelines and *P. falciparum*, is an open question that deserves further study.

**Conclusions:**

Clearly, there is a wealth of functional information to be gained from detailed manual genome annotation, yet the rapid increase in the number of available sequences means that most researchers will not have the time or resources to manually annotate all the sequence data they generate. We believe that efforts to maximize the amount of information obtained from automated annotation can help address the functional annotation deficit that most evolutionary biologists now face, and here demonstrate the value of such an approach.

**Electronic supplementary material:**

The online version of this article (doi:10.1186/s12864-017-3590-0) contains supplementary material, which is available to authorized users.

## Background

Mosquitos have been called the “deadliest animal in the world” [1], causing more than a million human deaths each year [2]. Much of this lethal impact can be attributed to malaria, which caused about 438,000 deaths in 2015 alone [3]. Though there are approximately 2,500 known species of mosquito, the transmission of *Plasmodium falciparum*, the parasitic protozoan responsible for the most lethal form of human malaria, depends on a single genus, *Anopheles* (Diptera: Culicoidea: Culicidae: Anophelinae). Within *Anopheles* there is significant variation in both vector competence (~70 of the 450 known anopheline species are *P. falciparum* vectors [4]) and vectorial capacity (the efficiency with which *P. falciparum* is transmitted to humans [5]). Here we use comparative genomics among *Anopheles* and closely related non-vector species to examine the genome content changes involved in vector competence and capacity.

The number of *Anopheles* species for which genome and transcriptome assemblies are available recently increased from five to 21, thanks to ambitious, multi-investigator efforts [6]. Such large, publicly available datasets are increasingly being generated, but the very scale of such data makes it impossible for a single researcher, or even a large consortium, to pursue all promising areas of inquiry. The hope is that researchers with diverse specialties will make use of the data to explore a realm of questions, but analytical barriers limit the usability of these datasets. In a Herculean effort, relying largely on manual annotation, Neafsey et al. [7] recently used the *Anopheles* data set, plus additional data, to examine the evolution of genes in categories relevant to vectorial capacity. Their analyses involved 120 scientists at 72 institutions, divided into more than 20 focal areas, and resulted in a wealth of new information about *Anopheles* genomes.

Unfortunately, such intense manual annotation is beyond the resources of most researchers. The persistent and growing gap between the availability of sequenced genomes and the ability to assign functions to sequenced genes led us to explore ways to maximize the information content of automatic annotation using GO terms and other curated, publicly available resources (e.g., UniProt and KEGG). Because such annotation methods tend to provide broad information about functional categories rather than about specific genes, we designed a custom “slimming” strategy to partition GO terms into categories relevant to vectorial capacity.

We investigated the genome content dynamics encompassed by gain and loss of protein families at ten internal nodes in the anopheline phylogeny. Examining internal nodes allows us to test hypotheses about the common ancestors of these vectors and to establish the sequence of events in evolutionary scenarios for the interaction of the vector with the parasite. Since the arms race between mosquitos and *Plasmodium spp*. parasites is millions of years old, the reconstructed ancestral steps that led to current genome content of these species are a critical, but so far missing, aspect of our understanding of vector capacity [8]. Strong evidence exists that mosquito defenses are an important barrier that *P. falciparum* must overcome [9, 10], and the genetic traits responsible for present-day vector efficacy can best be understood in an evolutionary context. Examination of the evolutionary timing and phylogenetic pattern of genome content changes (protein family gains and losses) in anophelines could shed light on the dramatic expansion in *P. falciparum*’s geographical range some 10,000 years ago, in concert with the spread of human agricultural societies [11].

Research on the evolution of vectorial capacity has typically focused on a set of well-understood functional categories (described in detail in Methods). In taking a bottom-up approach, we circumvent the bias that is inherent in a candidate-based approach. We portioned GO terms associated with gained/lost protein families according to MosquitoSlim, our custom slimming strategy, and analyzed any category that showed evidence of accelerated or significantly over-represented gain or loss. In this manner, we came to focus on a previously unexplored functional category relevant to vector competence: Nucleotide metabolism.

## Methods

### Identification of protein families gained/lost at internal nodes

**Fig. 1:**
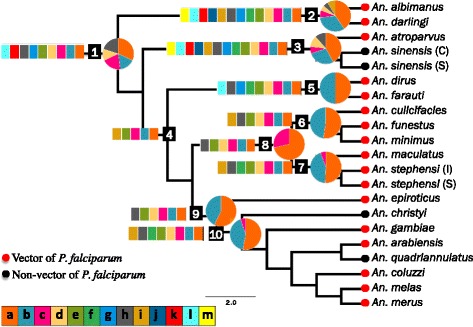
Anopheline phylogeny with gains, enriched gains, and vector status shown. Gains in MosquitoSlim categories are shown in *bars*, with each color present indicating that at least one gain in that category was detected at the associated node, and each color absent indicating a lack of gains in that category. Enriched gains for the nine nodes with enriched GO terms are shown in *pie charts*. The size of each slice represents the number of gained protein families associated with an enriched term in the respective MosquitoSlim subcategory. Vector status is shown by colored circles at the tips of the tree. Vector status information obtained from the Malaria Atlas Project (http://www.map.ox.ac.uk/map/) and references therein. Internal nodes KEY: 1. The subfamily Anophelinae; 2. *Nyssorhynchus*; 3. *Anopheles*; 4. *Cellia*; 5. *Neomyzomyia*; 6. *Myzomyia*; 7. *Neocellia*; 8. *Myzomyia* + *Neocellia*; 9. *Pyretophorous*; 10. The *gambiae* complex. MosquitoSlim KEY: *a* Related to nucleic acid metabolism, *b* Metabolic process, *c* Innate immunity, *d* Cell cycle, *e* Binding, *f* Cuticular protein, *g* Chemosensation, *h* Other, *i* Cellular component biogenesis *j* Cellular membrane, *k* Insecticide resistance, *l* Peptide hormones, *m* Epigenetic

We used classical phylogenetic reconstruction approaches to characterize gains and losses at the internal nodes in the anopheline phylogeny [12]. We first clustered proteins from 21 Anophelinae taxa and four outgroups into families by conducting all-against-all BLASTP searches with an e-value cutoff of e^−75^ followed by single-linkage clustering [13, 14]. As described in Rosenfeld et al. [12], we selected this e-value by comparing the ortholog group counts from values ranging from e^−5^ to e^−200^ and choosing the value that gave the maximum number of informative characters for all species and strains. To identify protein families gained or lost at each node, we used the “apolist” command in PAUP* [15] on the anopheline tree in Fig. 1 (default ACCTRANS setting for character reconstruction). We focused on the following ten nodes in the Anophelinae tree (node identifiers as used in Fig. 1 and throughout this communication): Anophelinae (node 1), *Nyssorhynchus* (node 2), *Anopheles* (node 3), *Cellia* (node 4), *Neomyzomyia* (node 5), *Myzomyia* (node 6), *Neocellia* (node 7), *Myzomyia* + *Neocellia* (node 8), *Pyretophorus* (node 9), and the *gambiae* species complex (node 10). Our protein family gain/loss set includes only families that were gained or lost at a single node, and singleton clusters were not analyzed. Protein sequences for *Drosophila melanogaster* were obtained from Ensembl (http://useast.ensembl.org) [16], sequences for all other species were obtained from VectorBase (https://www.vectorbase.org) [17]. See Additional file 1: Table S1 for list of taxa included.

### Gene Ontology (GO) mapping

After identifying the protein families gained or lost at each node, we chose a single representative sequence for each family and used several strategies to assign Gene Ontology (GO) terms to each sequence. While many sequences had already been assigned GO terms through annotation efforts at VectorBase using Maker [18] and WebApollo [19], some had not. For these sequences, we conducted BLASTP searches against a custom database containing all RefSeq proteins belonging to “Diptera” (Taxonomy ID: 7147; sequences were downloaded in late December, 2015; all parameters at default settings). Sequences that had no hit, or whose hit lacked a GO assignment, were blasted against the entire RefSeq nr database. We also conducted InterProScan 5 analyses [20] and added the resulting GO assignments to our data. The BLAST and InterProScan results were imported to Blast2GO [21], where we mapped them to GO terms. Sequences that lacked a GO term at this point were subjected to additional analysis using FFPred 2.0, a homology-independent tool for prediction of GO terms [22]. We filtered the FFPred results to only include GO predictions whose posterior probability was greater than or equal to 0.9 and whose underlying support vector regression models were classified as highly reliable. See Additional file 2: Figure S1 for flowchart of methods.

### MosquitoSlim classification of Gene Ontology (GO) terms

To assess the relevance of protein family gain/loss events at internal nodes to anopheline competence and capacity as vectors of *P. falciparum*, we created MosquitoSlim, a custom GO slimming system to partition GO terms into discrete categories. While a generic GO Slim classification is effective at consolidating GO terms into a biologically understandable framework, it does not group GOs according to functions of interest to a particular research question. Following the guidelines provided by the GO Consortium [23], our goal was to map as many of the anopheline GO terms as possible and partition them into categories relevant to vector status. We started with eight functional categories whose importance for vectorial competence and capacity are well understood, then added high level GO terms as required to map all the GO terms (Additional file 1: Table S2). MosquitoSlim includes 1,294 GO terms grouped into 18 categories--see Additional file 1: Table S2 for categories, term counts and sources used, Additional file 1: Table S3 for all the GO terms in MosquitoSlim. The full set of Anophelinae sequences included 241,878 proteins with GO annotation, and 95% of the assigned GO terms mapped to MosquitoSlim. The unmapped GO terms are shown in Additional file 1: Table S4.

Using the single occurrences count method in CateGOrizer [24], we assigned GO terms associated with gain/loss protein families to MosquitoSlim categories. We counted the number of protein families gained or lost in each category by taking all the GOs that mapped to a given MosquitoSlim category and counting the number of protein families that had those GO terms.

### InterPro assignment

Some gain/loss protein families were anonymous after being clustered. To determine which InterPro domains, families, or active sites the gain/loss families represented, we ran InterProScan 5 for a representative sequence from each cluster. Because InterPro signatures are often based on structural or sequence similarity that may not reveal a specific function, we used InterPro2GO [25] to assign the InterPro groups to GO terms to get additional information about protein function.

### KEGG mapping

We examined gain/loss families at the sequence level to determine the KEGG pathways to which they belonged. We used BlastKOALA (KEGG Orthology And Links Annotation) [26], KEGG’s internal annotation tool, to BLAST a single representative from each gain/loss family against the KEGG GENES database for the subfamily Culicidae (Taxonomy ID: 7157). Sequences with hits to the database were assigned to KO groups, and these were mapped to their respective KEGG Pathways.

### Assessment of functional enrichment

To test for over-representation of functional categories in the gain/loss dataset, we ran two-sided Fisher’s exact tests (FDR adjusted *p*-value < 0.05) to identify GO terms that were over- or under-represented in a test set of annotated proteins relative to a reference set. First, to determine how the Anophelinae as a whole differ from the Culicinae, we compared the set of annotated proteins from each subfamily to those of *D. melanogaster*. To detect enrichment in categories that might contribute to the anophelines’ status as vectors of *P. falciparum*, we used the Anophelinae proteins as our test set and Culicinae proteins as the reference set. The Culicinae are blood feeders and vector many important diseases, but are refractory to *P. falciparum*. We examined GO enrichment in the gain/loss protein set relative to *D. melanogaster* and relative to the Culicinae.

We also examined GO enrichment explicitly in regard to vector competence. Anopheline non-vectors of *P. falciparum* occur in Node 2 (*An. sinensis*) and Node 10 (*An. christyi* and *An. quadriannulatus*). Because these nodes also contain vector species, we grouped anophelines by vector competence rather than node to compare all *P. falciparum* vectors to all non-vectors. To avoid any bias arising from the fact that *An. gambiae* has far more annotated proteins than any other vector species, we conducted enrichment tests with and without this species in the test set. Finally, to look at vector-specific enrichment in the gained protein families, we identified all the orthologues and paralogues of protein families gained in Node 10 (the *gambiae* complex, which includes five vector species and two non-vectors) and looked for enrichment in Node 10 vectors versus non-vectors.

## Results

### General gain/loss patterns

There were 17,966 proteins in our presence/absence matrix. Using an e-value cutoff of e^−75^, we clustered these into 10,726 non-singleton families, each representing a distinct set of evolutionarily related sequences. Five hundred and fifty-nine families met our criteria of gain or loss at only one node.

Gains (531) were far more common than losses (531 versus 28), and this pattern applied to all ten nodes examined (Table 1). The number of gain/loss events, however, varied widely between nodes: *Nyssorhynchus* (node 2) had 152 gains and nine losses, while *Pyretophorus* (node 9) had just 13 gains and three losses.

**Table 1:** Summary of protein family gains and losses by node with annotation counts shown

Node	Type	Number of protein families	Number with GO term	GO terms	InterPro families	InterPro2GO terms	KEGG Orthologues
Anophelinae (Node 1)	Gain	15	12	53	5	17	3
	Loss	7	7	12	NA	NA	NA
	Enriched gain	NA	10	47	NA	NA	NA
Nyssorhynchus (Node 2)	Gain	152	123	105	70	61	28
	Loss	9	9	15	NA	NA	NA
	Enriched gain	NA	68	56	NA	NA	NA
Anopheles (Node 3)	Gain	91	70	62	33	31	1
	Loss	3	3	3	NA	NA	NA
	Enriched gain	NA	38	38	NA	NA	NA
Cellia (Node 4)	Gain	10	7	11	9	9	1
	Loss	1	1	1	NA	NA	NA
	Enriched gain	NA	0	0	NA	NA	NA
Neomyzomia (Node 5)	Gain	57	44	42	25	24	5
	Loss	4	3	6	NA	NA	NA
	Enriched gain	NA	26	45	NA	NA	NA
Myzomia (Node 6)	Gain	39	30	23	15	14	2
	Loss	1	1	3	NA	NA	NA
	Enriched gain	NA	13	26	NA	NA	NA
Neocellia (Node 7)	Gain	83	70	42	43	37	7
	Loss	0	0	0	NA	NA	NA
	Enriched gain	NA	42	49	NA	NA	NA
Myzomia + Neocellia (Node 8)	Gain	22	20	24	9	9	3
	Loss	0	0	0	NA	NA	NA
	Enriched gain	NA	5	3	NA	NA	NA
Pyretophorus (Node 9)	Gain	13	10	24	3	5	1
	Loss	3	3	12	NA	NA	NA
	Enriched gain	NA	6	12	NA	NA	NA
gambiae complex (Node 10)	Gain	49	43	30	19	19	7
	Loss	0	0	0	NA	NA	NA
	Enriched gain	NA	26	48	NA	NA	NA
Cumulative	Gain	531	429	196	145	64	58
	Loss	28	27	43	NA	NA	NA
	Enriched gain	NA	234	75	NA	NA	NA

### Assignment of GO terms

Of the 559 gain/loss families, 47% were mapped to GO terms using either BLAST or InterProScan. In contrast, 65% of the entire set of 17,966 proteins had GO terms at this stage, implying that many of the gain/loss sequences are “novel” in that they lack annotated homologues and may potentially represent anopheline-specific sequences. Further evidence for the novelty of the gain/loss set can be seen in the distribution of e-value and HSP/Hit coverage percent (Additional file 3: Figure S2 and Additional file 4: Figure S3), where higher e-values and lower coverage of the hit sequence to RefSeq proteins imply that the gain/loss sequences are, on average, more divergent than the anopheline protein set as a whole.

Analysis with FFPred allowed us to assign GOs to an additional 213 gain/loss families, bringing the final proportion of GO-annotated proteins to 82% for the gain/loss set. A total of 970 GOs, representing 274 distinct GO terms, were assigned to the gain/loss families. Of these, 75 GO terms were enriched in at least one node. GO terms and the frequency with which they were associated with gains, losses, and enrichment are shown in Additional file 1: Table S5.

Because our functional analyses are primarily GO-based, we examined the evidence upon which our GO assignments were based. As is typical for non-model organisms, the majority (79%) were inferred from electronic annotation (IEA), while only 5% were based on experimental confirmation (see Additional file 5: Figure S4 for the distribution of evidence codes assigned). We assessed the quality of the GO assignments by comparing the GO annotation scores for all anopheline proteins to those for the gain/loss families. As shown in Additional file 6: Figure S5, annotation scores were noticeably lower for the gain/loss set, consistent with our finding of less-significant e-values for these proteins.

### Functional classification of gain, loss, and GO enrichment

The GO terms associated with gain/loss families were classified into 18 MosquitoSlim categories (Table 2). Gains and losses were similarly distributed amongst categories (Additional file 7: Figure S6), with over 90% of the families falling into just seven categories: *Related to nucleic acid metabolism*, *metabolic process*, *top 20% EvoRate*, *other*, *innate immunity*, *cell cycle*, and *binding*.

**Table 2:** Summary of gains (G), losses (L) and enriched gains (E) at each node by MosquitoSlim category

Node	Type	Related to nucleotide metabolism	Metabolic process	Top 20% EvoRate	Other	Innate immunity	Cell cycle	Binding	Cuticular protein	Chemosensation	Cellular component biogenesis	Bottom 20% EvoRate	Cellular membrane	Insecticide resistance	Peptide hormone	Top 20% dN/dS	Epigenetic	Bottom 20% dN/dS	Salivary
Anophelinae (Node 1)	Enriched	8	4	5	5	5	3	0	0	0	0	0	0	0	0	0	0	0	0
	Gain	10	6	8	9	7	8	5	0	2	0	0	0	1	1	1	0	0	0
	Loss	7	1	2	0	2	1	2	1	0	0	0	3	0	0	0	0	0	0
Nyssorhynchus (Node 2)	Enriched	38	31	29	5	7	5	0	0	0	8	0	0	0	0	0	0	0	0
	Gain	97	53	65	18	33	22	18	16	7	10	10	4	4	2	2	1	0	0
	Loss	8	6	4	2	1	1	2	1	0	0	0	1	0	0	0	0	0	0
Anopheles (Node 3)	Enriched	29	19	16	8	5	8	0	0	0	0	0	0	0	0	0	0	0	0
	Gain	54	31	38	17	21	13	6	3	8	2	3	4	2	1	0	1	0	0
	Loss	2	1	1	0	0	0	0	0	0	0	0	0	0	0	0	0	0	0
Cellia (Node 4)	Gain	5	4	1	0	2	0	2	0	0	1	0	0	0	0	0	0	0	0
	Loss	1	0	1	0	0	0	0	0	0	0	0	0	0	0	0	0	0	0
Neomyzomia (Node 5)	Enriched	17	17	14	0	0	0	0	0	0	0	0	0	0	0	0	0	0	0
	Gain	36	23	30	6	10	5	8	1	2	0	1	0	0	1	1	0	0	0
	Loss	2	0	0	2	0	2	0	0	1	0	0	0	0	0	0	0	0	0
Myzomia (Node 6)	Enriched	11	10	7	0	0	0	0	0	0	0	0	0	0	0	0	0	0	0
	Gain	25	11	6	4	1	4	7	0	0	1	1	0	0	0	0	0	1	0
	Loss	1	0	1	0	0	0	1	0	0	0	0	0	0	0	0	0	0	0
Neocellia (Node 7)	Enriched	27	23	15	0	3	0	0	0	0	0	0	0	0	0	0	0	0	0
	Gain	58	32	12	15	5	10	13	7	0	3	0	0	0	0	0	0	0	0
Myzomia + Neocellia (Node 8)	Enriched	5	0	0	0	2	0	0	0	0	0	0	0	0	0	0	0	0	0
	Gain	12	7	4	4	2	2	4	0	0	0	0	0	0	0	0	0	0	0
Pyretophorus (Node 9)	Enriched	4	3	3	0	0	0	0	0	0	0	0	0	0	0	0	0	0	0
	Gain	9	6	1	2	1	2	2	0	0	0	0	0	0	0	0	0	0	0
	Loss	3	3	1	0	0	1	2	0	0	0	1	0	0	0	0	0	1	0
gambiae complex (Node 10)	Enriched	20	16	11	0	2	0	0	0	0	0	0	0	0	0	0	0	0	0
	Gain	39	24	8	11	3	9	8	4	0	1	2	0	0	0	0	0	0	0

**Fig. 2:**
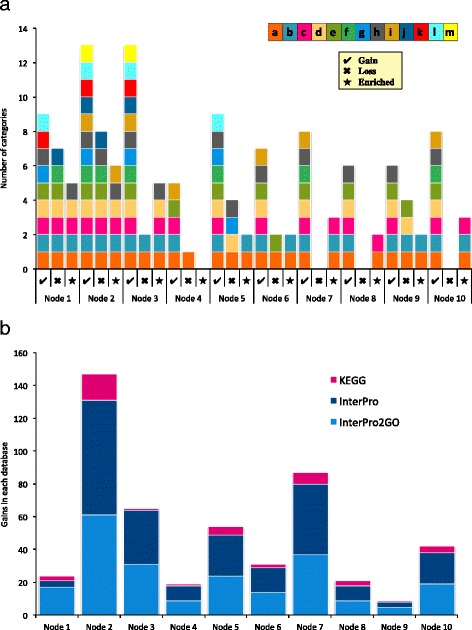
Protein family diversity. 2.1. Diversity in MosquitoSlim categories. For each node, gains, losses (if any), and enriched gains (if any) are shown. Each color present in the stacked column indicates that at least one protein family in that category was detected at the associated node, and each color absent indicates the lack of protein families in that category. MosquitoSlim KEY: ***a*** Related to nucleic acid metabolism, ***b*** Metabolic process, *c* Innate immunity, *d* Cell cycle, *e* Binding, *f* Cuticular protein, *g* Chemosensation, *h* Other, *i* Cellular component biogenesis *j* Cellular membrane, *k* Insecticide resistance, *l* Peptide hormones, *m* Epigenetic. 2.2. Diversity in other annotation methodologies. Each color represents a different database, and the height of each individual block in the column reflects the number of distinct categories. For example, Node 2 has protein family gains that were assigned to 16 different KEGG objects, 70 different InterPro groups, and 61 different InterPro2GO terms

Seventy-five GO terms from 234 gain families were enriched in comparison to the Culicinae reference in at least one node. The enriched GO terms were classified into seven MosquitoSlim categories, primarily *related to nucleic acid metabolism*, *metabolic process*, *top 20% EvoRate*, and *innate immunity*. Gains at nine out of the ten nodes examined had significantly enriched GO terms (Fig. 2). All of the enriched GO terms were associated with gains rather than losses, and almost all of the enriched terms were over-represented in the test set versus the Culicinae reference set. We do not discuss the under-represented families here, as interpretation of the biological significance of under-representation is problematic. The number of protein families with enriched GO terms at a given node was not correlated with the total number of gains at that node.

### InterPro assignment and InterPro2GO mapping

One hundred and fifty-eight of the gained protein families were assigned to one or more InterPro groups, while 373 gained families had no InterPro assignment. One hundred and forty-five InterPro groups were represented, and more than half of these were gained at only one node. Among the most commonly assigned InterPro groups were zinc finger domains and a pheromone/general odorant binding protein family. Full InterPro results are shown in Additional file 1: Table S6.

Ninety-six of the 145 InterPro groups mapped to InterPro2GO GO terms. A total of 110 InterPro2GO terms were found. While 66% of the InterPro2GO terms were associated with only one InterPro group, some terms included as many as 10 InterPro groups (Additional file 1: Table S7). The most frequently gained InterPro2GO terms were nucleic acid binding, metal ion binding, zinc ion binding, and odorant binding, all of which were associated with protein family gains at 7 of the 10 nodes examined.

### KEGG mapping of gained protein families

Fifty-eight gained protein families distributed across all 10 nodes were assigned to 40 KEGG Orthologues (KOs) in the Culicidae database. The proportion of protein families having a KO hit varied between nodes (Table 1) and was not correlated with the number of gains at a node. We mapped the KOs to their respective KEGG objects (pathways or reference hierarchies) and found that a few pathways predominated: 03010 Ribosome, 00190 Oxidative phosphorylation, and 00230 Purine metabolism/00240 Pyrimidine metabolism (see Additional file 1: Table S8 for all KEGG results). Where GO terms were available for the KOs, we mapped them to MosquitoSlim and found good agreement between the KEGG and MosquitoSlim classifications.

### Comparison to previous findings

An alternative method of assessing protein family expansion and contraction in the Anophelinae was recently used by Neafsey et al. [7], who focused on the primary sequence of proteins rather than presence/absence patterns. They analyzed protein family gains and losses by utilizing CAFE 3 [27], a program that uses proteins family information at the tips of trees to estimate gain/loss patterns. Such an approach does not take into account changes in genome content in common ancestors in the phylogenetic tree, and therefore cannot distinguish protein families that are repeatedly gained and lost from those that are associated with terminals in the tree. We have taken an explicit character reconstruction approach and restricted our set of gained or lost protein families to those that are unique to a single internal node.

While the goal of Neafsey et al.’s approach was to use the number of proteins in a family to understand organismal function, our goal was to use the number and kind of distinct protein families gained or lost at each internal node to understand the innovations at each node that might contribute to cladogenesis and functional diversification. Despite these differences, the clustering method we used produced about the same number of non-singleton clusters as Neafsey et al. reported (10,726 clusters in our analysis, 11,636 in Neafsey et al.’s). They assessed the number of proteins in each cluster that were present at each terminal node (i.e., family member counts per species), while we assessed when the cluster as a whole originated at ancestral nodes in the tree.

Neafsey et al. found a surprising lack of obvious vector-related evolution in the “usual suspect” categories, but they did report on expansion or contraction of specific InterPro protein families and on overall sequence divergence rates. We found gains of protein families with GO terms from Neafsey et al.’s top 20% evolutionary rate set at every node, confirming that sequence divergence is notably elevated in protein associated with these GO terms. This is expected given that most “gained” protein families presumably originate from duplication events followed by sequence divergence and neo-functionalization. An alternative pathway to “gained” families not involving duplication is for a functional gene to be silenced via sequence change and to then neo-functionalize. Although there is no standard cutoff for the amount of sequence divergence that is needed for a protein to take on a new function, clustering based on strict similarity can potentially reveal candidates for neo-functionalization.

In many cases, our clustering criteria (reciprocal Blast scores ≤ e^−75^) generated 2+ distinct clusters for a single InterPro family. For example, Neafsey et al. [7] reported expansion of IPR000618 (Insect cuticle protein) in *An. arabiensis* (Node 10), while we found distinct gains of this family at Nodes 2, 3, and 9. For other InterPro groups we found a single cluster, and for almost all the expansions discussed by Neafsey et al. we found that the expanded family originated quite early in the phylogeny and was not unique to the node where expansion is reported. For example, Neafsey et al. [7] found significant expansion of IPR004117 (Olfactory receptor, *Drosophila*) in *An. gambiae* (Node 10), while we localize the original gain of this family at Node 1. Finally, many of the InterPro groups discussed by Neafsey et al. [7] were not in our gain/loss set, implying that those protein families were not uniquely gained or lost at any node we examined. See Additional file 1: Table S9 for a full comparison.

### Analysis of anopheline biology using reconstructed gain/loss events

By mapping gains and losses to the node at which they occurred, we found that the broadest diversity of gains and losses happened early in the evolution of anophelines (Fig. 2). Surprisingly few of the gained families were in categories previously thought to contribute to vector competence and capacity, and many of these categories occurred only in early nodes: Protein families in the MosquitoSlim categories *epigenetic*, *insecticide resistance*, *peptide hormones*, and *chemosensation* were not gained beyond Node 5, and were not enriched in any node. Two canonical categories, however, did show gains in equal or greater numbers in late and early diverging nodes: *Cuticular proteins*, which contribute to insecticide resistance, were gained in Nodes 2, 3, 5, 7, and 10, while *innate immunity* proteins, considered a key aspect of anophelines’ resistance to *P. falciparum*, were gained in all nodes and enriched in six out of ten nodes. The most frequently gained and enriched category was *related to nucleotide metabolism*, which is discussed in detail below.

### Nucleotide metabolism—an unexpected component of vectorial capacity?

Nucleotides are vital cellular components, serving as the building blocks of nucleic acids, as energy transfer modules (e.g., ATP), and as components of cellular signaling networks (e.g. cAMP) [28]. *Plasmodium falciparum*, like almost all parasitic protozoa studied to date, are unable to synthesize purine nucleotides *de novo* [29], and genome sequencing of closely related parasitic protozoa has shown that they lack the purine synthesis genes found in other eukaryotes [30]. *Plasmodium falciparum* must therefore scavenge purine nucleotides or their precursors from its hosts. *Plasmodium falciparum*’s need for nucleic acids is especially acute in the life stages at which rapid cell division occurs: Male gamete development and sporozoite formation, which occur in the anopheline host, and liver-stage development and blood-stage asexual reproduction, which occur in the vertebrate host [31].

In humans, the mechanisms of purine salvage by *P. falciparum* in human erythrocytes have been investigated as a potential anti-malarial drug target [32], but the technical difficulty of culturing *P. falciparum* in a pseudo-mosquito environment has limited our knowledge of how *P. falciparum* acquires purines from its anopheline host. The purine metabolism pathways in the mosquito itself, however, have been examined [33]. Overall, the *Anopheles gambiae* pathway is quite similar to that of humans, and the *An. gambiae* genome contains orthologues of most known human purine metabolism genes [34]. In both humans and mosquitoes, the ability to salvage purine nucleosides through the enzymatic action of adenosine kinase (AdK; EC 2.7.1.20) distinguishes the host from the parasite. Adenosine kinase’s high affinity for the purine nucleoside adenosine limits the availability of adenosine to *P. falciparum* [35], and may act as an anti- *P. falciparum* defense.

**Fig. 3:**
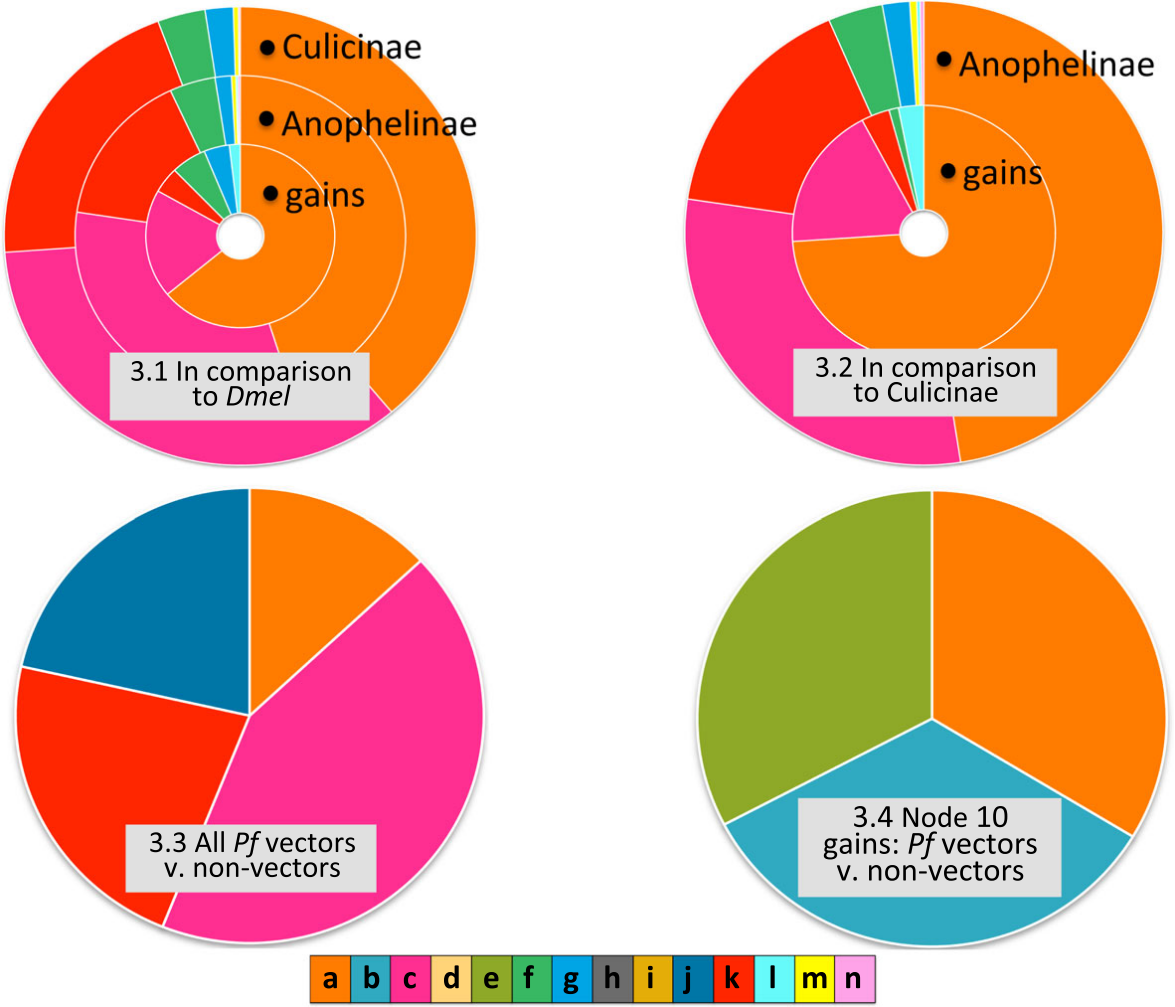
Functional enrichment in comparison to a reference protein set. 3.1. With all annotated *Drosophila melanogaster* proteins as the reference set, enrichment results are shown for: All annotated Culicinae (*A. aegypti* + *C. quinquefasciatus*) proteins (outer ring, labeled “Culicinae”); All annotated Anophelinae proteins (middle ring, labeled “Anophelinae”); Protein families gained in the ten anopheline nodes (inner ring, labeled “Gains”). 3.2. With all annotated Culicinae proteins as the reference set, enrichment results are shown for: All annotated Anophelinae proteins (outer ring, labeled “Anophelinae”); Protein families gained in the ten anopheline nodes (inner ring, labeled “Gains”). 3.3. With all annotated proteins from anopheline non-vectors of *Plasmodium falciparum* (*An. sinensis* (C), *An. sinensis* (S), *An. christyi*, and *An. quadriannulatus*) as the reference set, enrichment results are shown for all annotated proteins from anopheline vectors of *P. falciparum*. 3.4. Using only proteins that are orthologues or paralogues of protein families gained in Node 10 (the *gambiae* complex), enrichment results are shown for Node 10 vectors of *P. falciparum* in comparison to Node 10 non-vectors of *P. falciparum*. MosquitoSlim KEY: *a* Related to nucleic acid metabolism, *b* Metabolic process, *c* Innate immunity, *d* Cell cycle, *e* Binding, *f* Cuticular protein, *g* Chemosensation, *h* Other, *i* Cellular component biogenesis *j* Cellular membrane, *k* Insecticide resistance, *l* Peptide hormones, *m* Epigenetic, *n* Salivary
II. *Compared to other mosquitos, anophelines are enriched in the* related to nucleotide metabolism *category.* Here we used all Anophelinae proteins or the gained protein set as our test set and Culicinae as our reference. Enrichment in this comparison could suggest vector-related enrichment in anophelines that is not typical of mosquitos as a whole. As shown in Fig. 3, we found enrichment in the same categories as in the *D. melanogaster* comparison, except that the gain set was not enriched in *chemosensory* proteins.
III. *Compared to anophelines that do not vector P. falciparum, vectors are enriched in the* related to nucleotide metabolism *category.* We compared vectors to non-vectors in two ways. Using all annotated proteins from vector species as the test set, and non-vector species as the reference, we found enrichment in the categories *innate immunity*, *insecticide resistance*, *cellular membrane*, and *related to innate immunity* (Fig. 3). When we considered only the orthologues and paralogues of gained protein families from Node 10 (Fig. 3), we found that *gambiae* complex vectors of *P. falciparum* were enriched for a mostly different set of categories. *Related to nucleotide metabolism* was the only shared category, while *binding* and *metabolic process* were also enriched.
  Taken together, these results suggest that proteins related to the synthesis, degradation, and salvage of nucleotides are over-represented in anopheline genomes as a whole, and have been gained with disproportionate frequency throughout the anopheline phylogeny. At a gross level, anophelines appear to be as different from culicines as they are from *D. melanogaster*, implying that the enriched categories are not simply a characteristic of mosquitos in comparison to other dipterans. This pattern is even more noticeable in the set of protein families gained across the ten anopheline nodes we examined (Fig. 1, pies), suggesting that gains of protein families involved in nucleotide metabolism may have contributed to the divergence of anophelines from other mosquitos, and perhaps to their status as the only known vectors of *P. falciparum*.

Protein families whose annotations suggest involvement in the synthesis, degradation, and salvage of purines and pyrimidines were surprisingly common in the set of gained protein families we examined: More than 30% of the gained families had GO terms that mapped to the MosquitoSlim category *related to nucleotide metabolism*. To investigate whether this was an artifact of our methodology, we tested several hypotheses:

I. *Compared to other Diptera, all mosquitos are enriched in the* related to nucleotide metabolism *category*. Our reference set here was *D. melanogaster*, and our test set was either (a) all annotated proteins from *A. aegypti* and *C. quinquefasciatus*, (b) all annotated proteins from the Anophelinae, or (c) the set of gained proteins from all ten nodes. Most of the anopheline species in our data set are *P. falciparum* vectors, so shared enriched terms in the two test sets are presumably unrelated to vector competence, but may be related to the divergence of mosquitos from other dipterans. Any enriched terms found only in anophelines could point to vector-related enrichment.
  We found that Anophelinae and Culicinae had similar levels of enrichment in the r*elated to nucleotide metabolism* category and in many of the “usual suspect” categories thought to be involved in vectorial competence and capacity, notably *innate immunity*, *insecticide resistance*, *cuticular proteins*, *chemosensory proteins*, *epigenetic proteins*, and *salivary proteins* (Fig. 3). Only anophelines showed enrichment in *peptide hormones*. The set of gained proteins was enriched in most of the same categories, but in different proportions—in comparison to all anopheline proteins, the gain set had more enrichment in *nucleotide metabolism* and *peptide hormones*, and less in *innate immunity* and *insecticide resistance*. The gained proteins were not enriched in *salivary* or *epigenetic* proteins.

## Discussion

### Genome content analysis as a source of phylogenetic and functional inference

Since we examined several nodes in the anopheline tree, we are able to make statements about the kinds of changes that occur during the cladogenesis of taxa in the group. We find that *Nyssorhynchus* (node 2), a Neotropical subgenus containing many malaria vector species, has almost twice as many protein family gains/losses as any other group. While the status of this node as one of the earliest diverging of those examined might partially explain its relative richness, other early cladogenesis events involve very few gains. A more meaningful comparison is to examine the diversity, rather than the number, of gains and enriched GO terms over the anopheline phylogeny. In spite of having variable numbers of gains, we find that early-diverging nodes show the greatest diversity of gains, and this diversity is reflected in their enriched GO terms (Fig. 1, bars). This same pattern was seen in the other annotation methods: In the KEGG, InterPro, and InterPro2GO results, Nodes 1, 2, and 3 together represented as much diversity as the other nodes combined.

### The evolution of vector competence

Gains of protein families in many of the categories expected to contribute to vector competence and capacity were found almost exclusively in the earliest diverging taxa, suggesting that the protein families responsible for the anophelines’ ability to tolerate and transmit *P. falciparum* were in place soon after the split between Culicinae and Anophelinae. If this is so, subsequent expansions and contractions may represent adaptive tinkering as anophelines seek to maximize their fitness in the presence of *P. falciparum* parasites. While there is some tendency to assume that protein family expansion in a given taxa is a measure of its functional importance in that taxa, we show here that most expansions are of protein families gained very early in the anophelines’ evolutionary history. We feel it is erroneous to assume that expansion of a protein family in, for example, an extremely virulent vector like *An. gambiae* necessarily implies that protein family expansion has contributed to vector capacity. Instead, we suggest that the many early gains of usual suspect (and other) protein families laid the foundation for the anophelines’ role as *P. falciparum* vectors.

The competence and capacity of *P. falciparum* vectors do not appear to be phylogenetically constrained within the Anophelinae. The distribution of non-vectors (Fig. 1) suggests that vector competence is an ancestral trait that has been lost at least twice, in *Anopheles* (Node 3) and in the *gambiae* complex (Node 10). Within the *gambiae* complex, the species *An. gambiae* and *An. quadriannulatus* diverged as recently as 2 mya [36], yet *An. gambiae* is the principal vector responsible for *P. falciparum* transmission to humans while *An. quadriannulatus* is largely refractory to *P. falciparum* [37]. Neither the protein family gains and losses at internal nodes that we present here, nor the species-specific protein family expansions and contractions reported by Neafsey et al. [7], appear to explain the observed distribution of vectors in the Anophelinae. Instead, we suggest that a previously unexamined component of vector biology, anopheline nucleotide metabolism, may contribute to the anophelines’ unique status as *P. falciparum* vectors.

While the fitness effects of nucleotide co-option by *P. falciparum* parasites on their anopheline hosts are not yet known, our results suggest that anopheline genomes may be responding to selection pressure exerted by *P. falciparum*. Nucleotide co-option by *P. falciparum* parasites could mimic the effect of mutations to genes involved in nucleotide metabolism. In humans, defects in the purine metabolism system are associated with a range of diseases, some of which are lethal, and mutations that result in improper nucleotide balance can limit the efficacy of nucleotide salvage in response to DNA damage [38]. While such fitness implications are still to be determined, the essential nature of purine availability for successful *Plasmodium* development and transmission make nucleotide metabolism an interesting trait underlying vectorial capacity, and the patterns we report here suggest that anopheline vectors of *P. falciparum* have expanded their repertoire of proteins related to nucleic acid metabolism. Whether this response is defensive, in an attempt to redress improper nucleotide balance resulting from *P. falciparum* infection, or perhaps symbiotic, resulting from an as-yet-unknown mutualism between anophelines and *P. falciparum*, is an open question that deserves further study.

Because we relied on electronic annotation to classify protein families into functional categories, our findings are quite broad and certainly don’t point to specific amino acid sequences as explanatory. While GO-based inferences are unlikely to be incorrect, they do tend to be quite general. Thus, while our annotation process might end at calling a sequence “integral component of membrane” (GO:0016021), manual annotation might refine this to “Cytochrome c oxidase subunit 1.” Clearly, there is a wealth of functional information to be gained from detailed manual approaches, yet the rapid increase in the number of available sequences means that most researchers will not have the time or resources to manually annotate all the sequence data they generate. We believe that efforts to maximize the amount of information obtained from electronic annotation can help address the functional annotation deficit that most evolutionary biologists now face.

## Conclusions

We have used genome content analysis of anopheline vectors of *P. falciparum* mosquitos to follow the loss and gain of protein families over the evolutionary history of this group. To address the lack of experimentally validated functional annotations for most of the proteins investigated, we employed a custom GO slimming tool, MosquitoSlim, to analyze the relevance of gained and lost protein families to the unique status of anophelines as *P. falciparum* vectors. Using ancestral reconstruction methods, we conclude that anopheline nucleotide metabolism may be of unexpected importance in Anophelinae-*P. falciparum* interactions. The fitness effects of nucleotide co-option by *P. falciparum* parasites on their anopheline hosts are not yet known, and our results suggest that this question deserves further study.

## Additional files

Additional file 1: Table S1. Taxa analyzed and sources of sequence data. **Table S2.** MosquitoSlim categories, GO term counts and source of GO terms. **Table S3.** GO terms included in MosquitoSlim. **Table S4.** GO terms that did not map to MosquitoSlim. **Table** **S5.** GO terms and the frequency with which they were associated with protein families gained, lost, and enriched. **Table S6.** InterPro IDs of gained protein families. **Table S7.** InterPro2GO mapping of gained InterPro families. **Table S8.** KEGG mapping of gained protein families. **Table S9.** Comparison with the results of Neafsey et al. [7]. (XLSX 128 kb)
Additional file 2: Figure S1. Flowchart of methods used to identify and annotate gain/loss protein families. (PDF 38 kb)
Additional file 3: Figure S2. The distribution of e-values obtained in BLASTP searches with two different query sets: All anopheline proteins except for the gain/loss set versus only the protein families in the gain/loss set. (PDF 44 kb)
Additional file 4: Figure S3. The distribution of HSP/Hit coverage percents obtained in BLASTP searches with two different query sets: All anopheline proteins except for the gain/loss set versus only the protein families in the gain/loss set. (PDF 41 kb)
Additional file 5: Figure S4. The evidence codes assigned to GOs associated with anopheline gain/loss proteins. (PDF 328 kb)
Additional file 6: Figure S5. GO annotation scores for two different gene sets: All anopheline proteins except for the gain/loss set versus only the protein families in the gain/loss set. (PDF 41 kb)
Additional file 7: Figure S6. Number of protein families gained (outer ring) and lost (inner ring) in each MosquitoSlim category. Counts are cumulative across all ten nodes. (PDF 107 kb)

## Abbreviations

Ae: Aedes
An: Anopheles
GO: Gene ontology
