# Supplementary material for: Genome content analysis yields new insights into the relationship between the human malaria parasite Plasmodium falciparum and its anopheline vectors
Source: BMC Genomics. 2017 Feb 27;18:205. doi: 10.1186/s12864-017-3590-0 (PMC5327517; doi:10.1186/s12864-017-3590-0)
Supplement: Additional file 2: Figure S1. — Flowchart of methods used to identify and annotate gain/loss protein families. (PDF 38 kb) [file 12864_2017_3590_MOESM2_ESM.pdf]

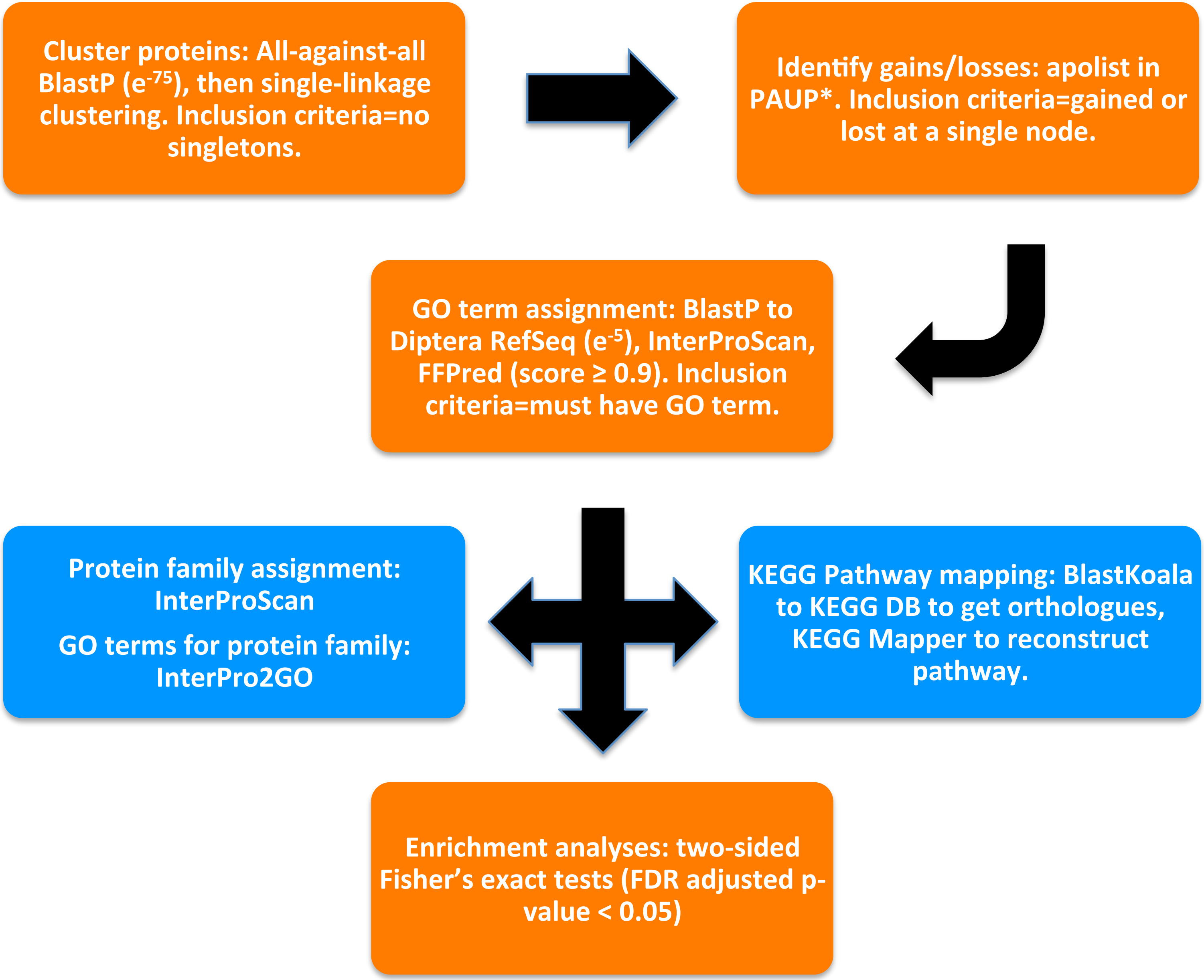
Cluster proteins: All-against-all BlastP (e^{-75}), then single-linkage clustering. Inclusion criteria=no singletons.
Identify gains/losses: apolist in PAUP*. Inclusion criteria=gained or lost at a single node.
GO term assignment: BlastP to Diptera RefSeq (e^{-5}), InterProScan, FFPred (score ≥ 0.9). Inclusion criteria=must have GO term.
Protein family assignment: InterProScan
GO terms for protein family: InterPro2GO
KEGG Pathway mapping: BlastKoala to KEGG DB to get orthologues, KEGG Mapper to reconstruct pathway.
Enrichment analyses: two-sided Fisher's exact tests (FDR adjusted p-value < 0.05)
