# Supplementary figures and images for: Genome content analysis yields new insights into the relationship between the human malaria parasite Plasmodium falciparum and its anopheline vectors
Source: BMC Genomics. 2017 Feb 27;18:205. doi: 10.1186/s12864-017-3590-0 (PMC5327517; doi:10.1186/s12864-017-3590-0)

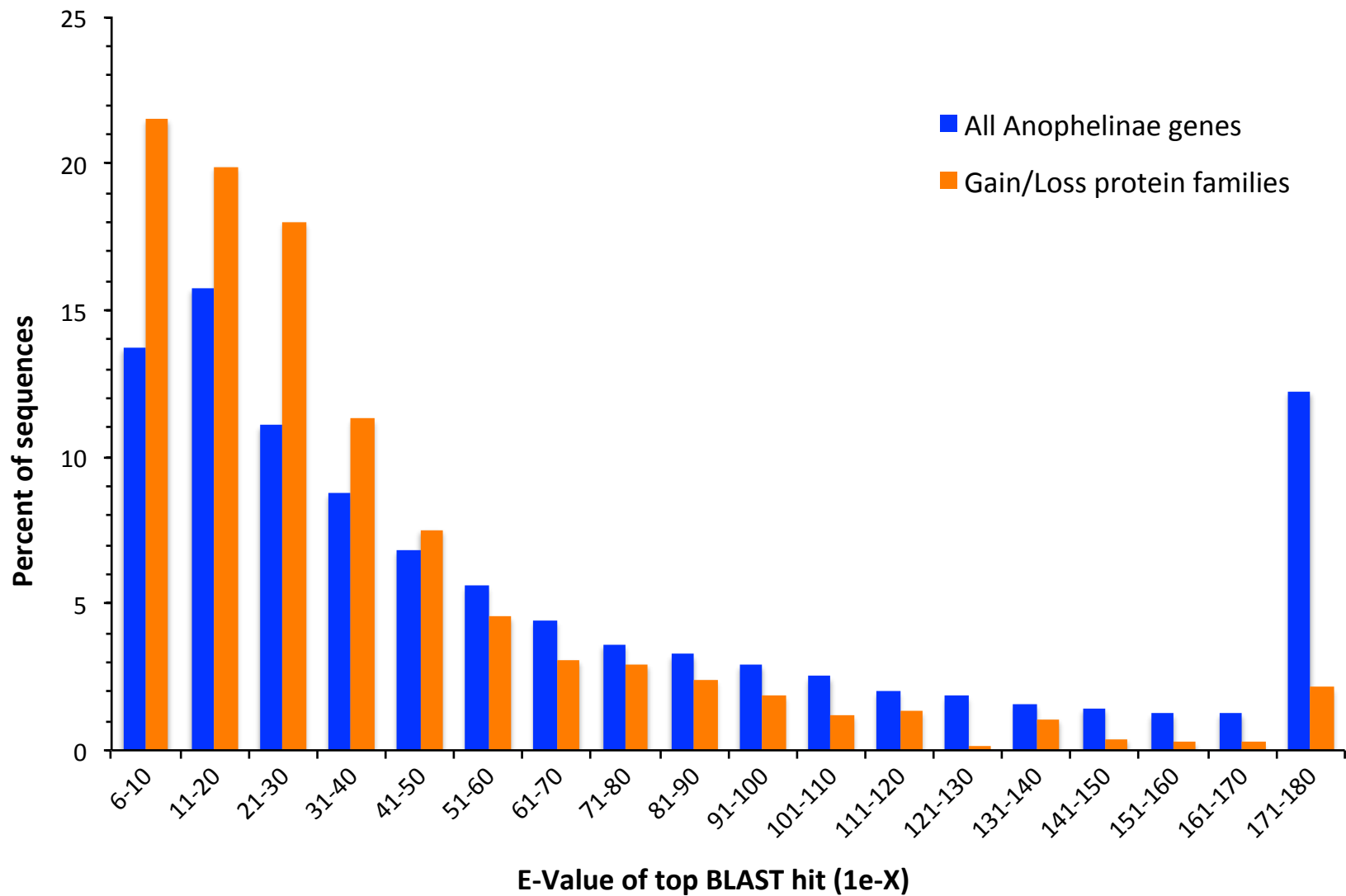

Supplement: Additional file 3: Figure S2. — The distribution of e-values obtained in BLASTP searches with two different query sets: All anopheline proteins except for the gain/loss set versus only the protein families in the gain/loss set. (PDF 44 kb) [file 12864_2017_3590_MOESM3_ESM.pdf]

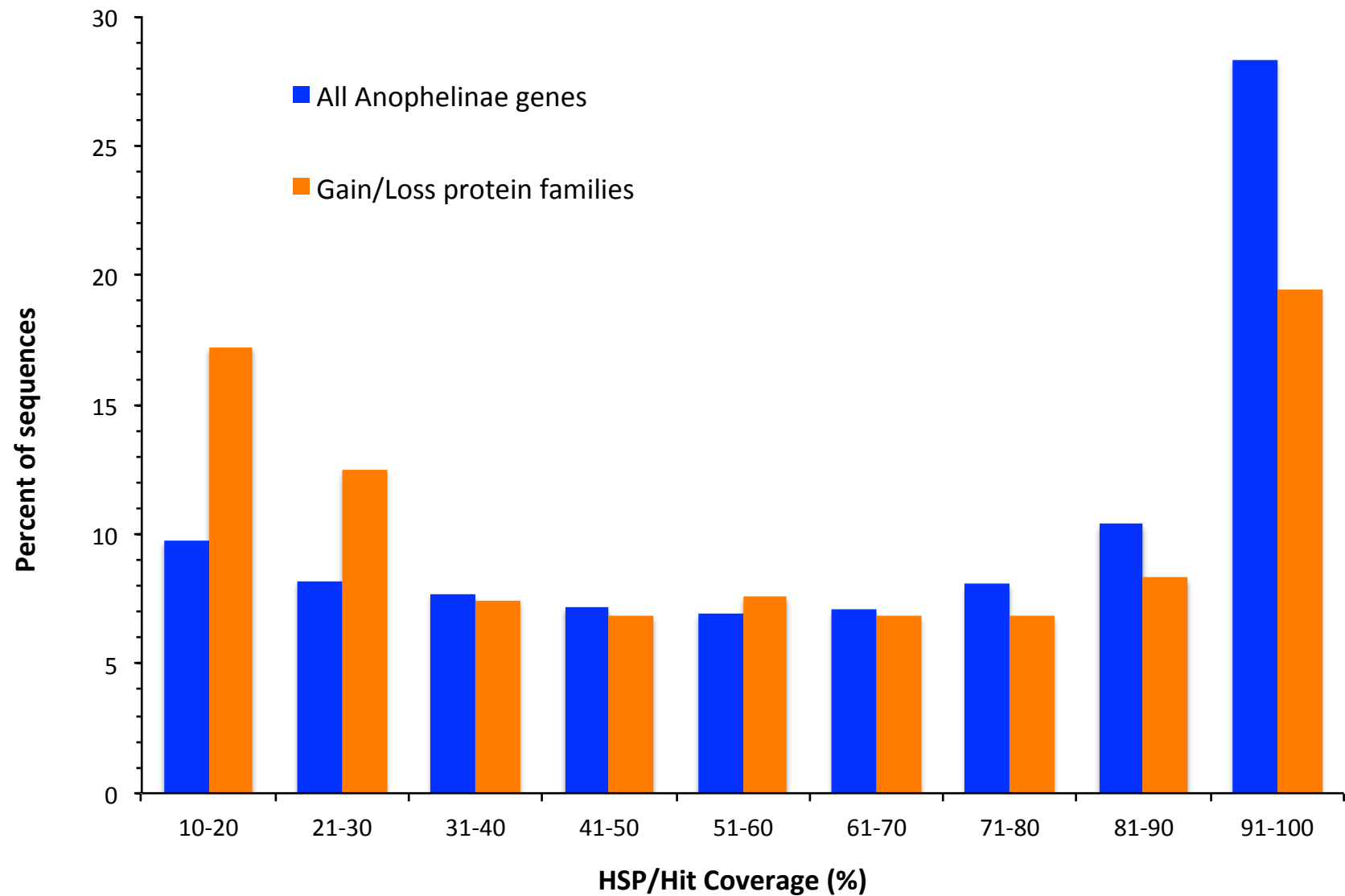

Supplement: Additional file 4: Figure S3. — The distribution of HSP/Hit coverage percents obtained in BLASTP searches with two different query sets: All anopheline proteins except for the gain/loss set versus only the protein families in the gain/loss set. (PDF 41 kb) [file 12864_2017_3590_MOESM4_ESM.pdf]

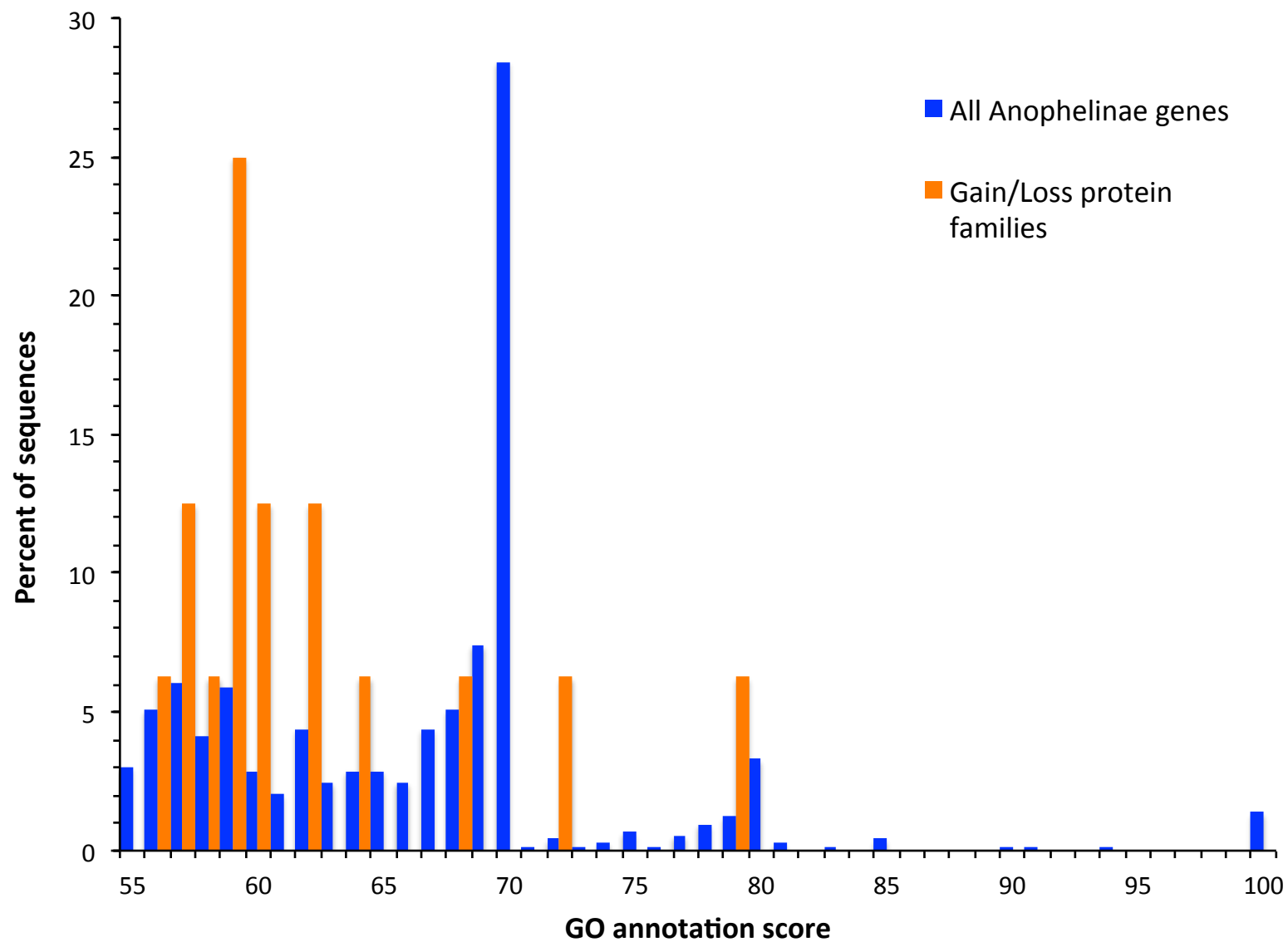

Supplement: Additional file 6: Figure S5. — GO annotation scores for two different gene sets: All anopheline proteins except for the gain/loss set versus only the protein families in the gain/loss set. (PDF 41 kb) [file 12864_2017_3590_MOESM6_ESM.pdf]

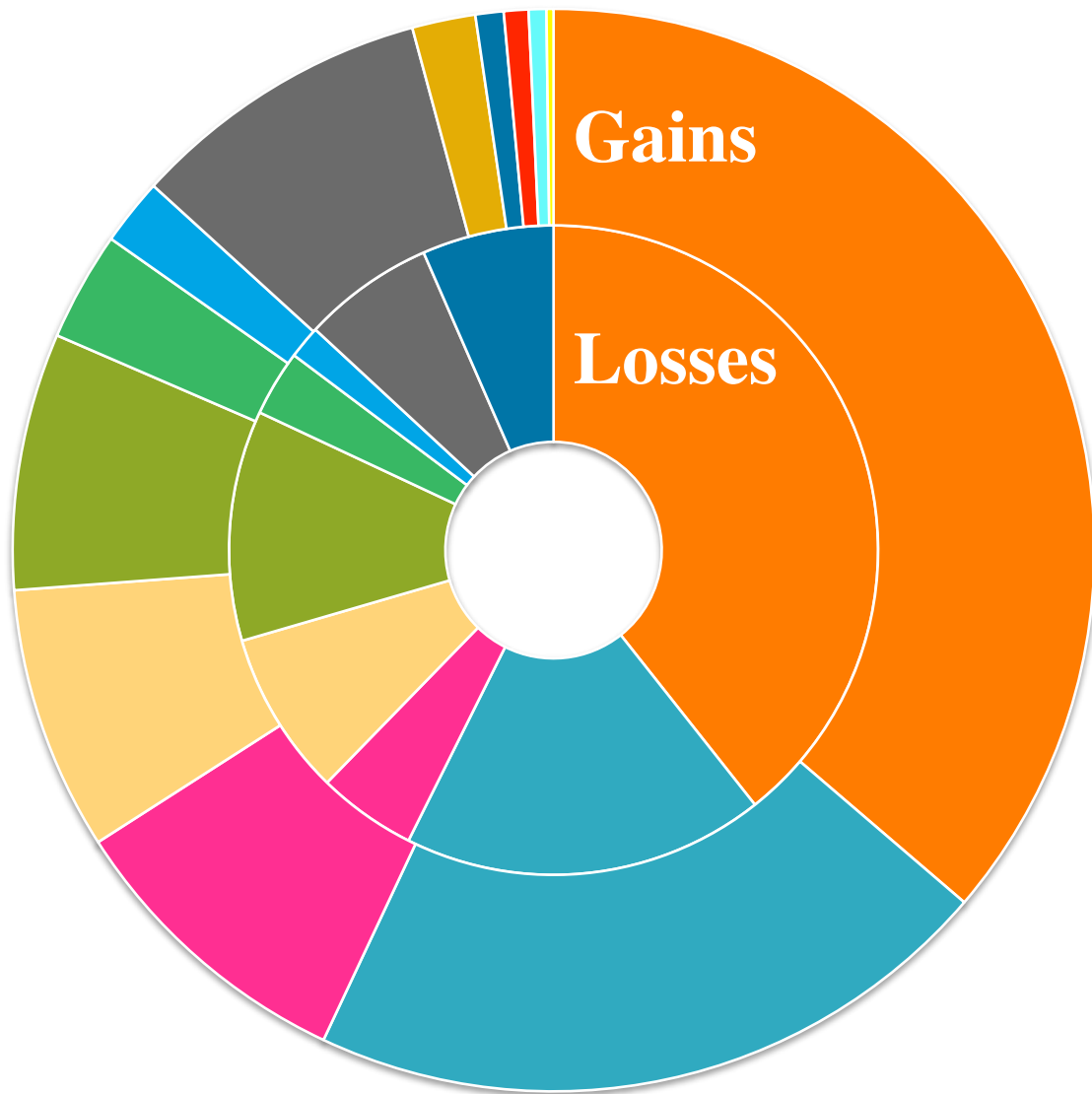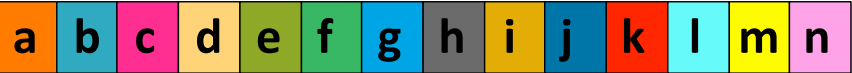

Supplement: Additional file 7: Figure S6. — Number of protein families gained (outer ring) and lost (inner ring) in each MosquitoSlim category. Counts are cumulative across all ten nodes. (PDF 107 kb) [file 12864_2017_3590_MOESM7_ESM.pdf]
