# Supplementary material for: Genome content analysis yields new insights into the relationship between the human malaria parasite Plasmodium falciparum and its anopheline vectors
Source: BMC Genomics. 2017 Feb 27;18:205. doi: 10.1186/s12864-017-3590-0 (PMC5327517; doi:10.1186/s12864-017-3590-0)
Supplement: Additional file 5: Figure S4. — The evidence codes assigned to GOs associated with anopheline gain/loss proteins. (PDF 328 kb) [file 12864_2017_3590_MOESM5_ESM.pdf]

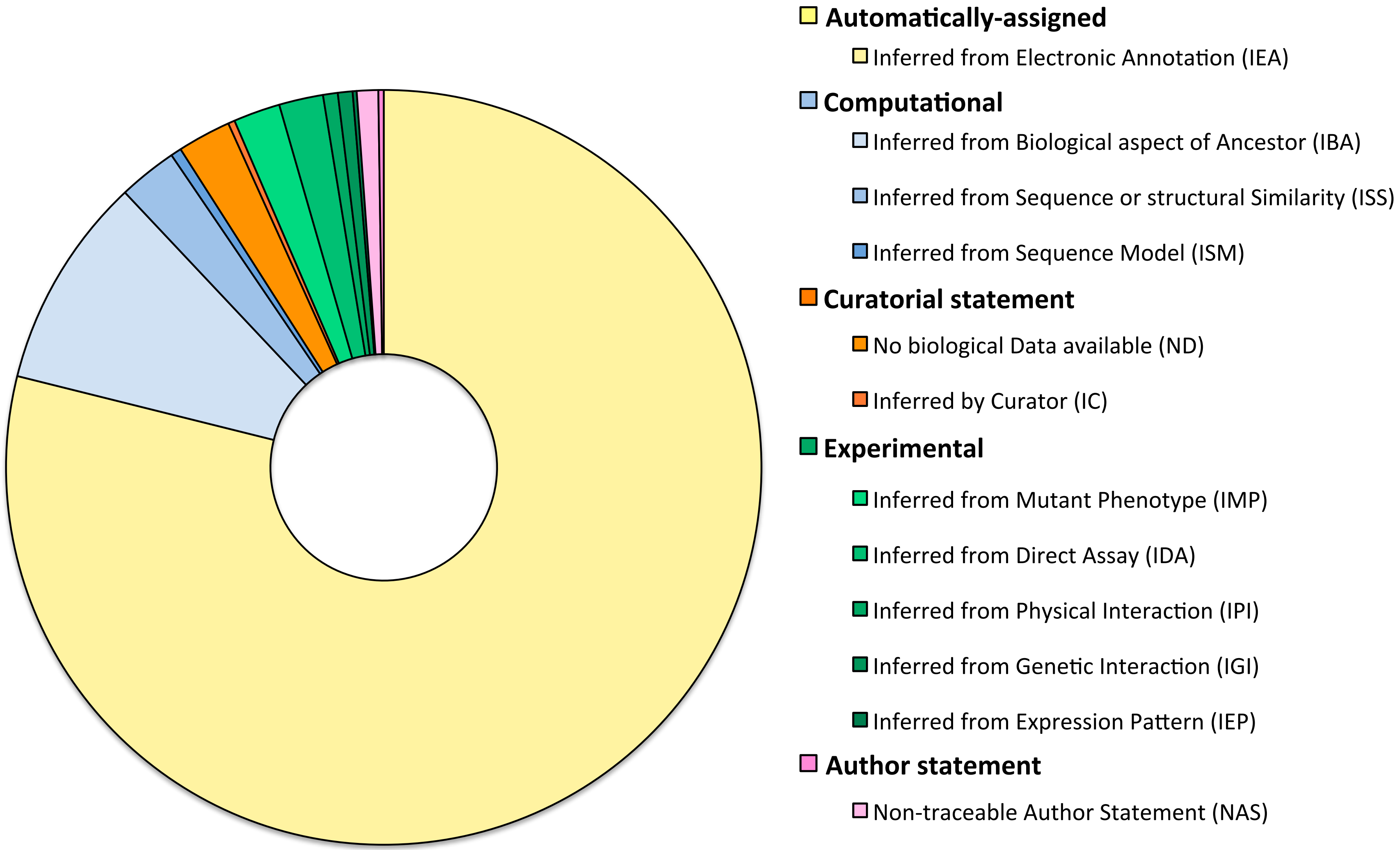

Automatically-assigned
Inferred from Electronic Annotation (IEA)
Computational
Inferred from Biological aspect of Ancestor (IBA)
Inferred from Sequence or structural Similarity (ISS)
Inferred from Sequence Model (ISM)
Curatorial statement
No biological Data available (ND)
Inferred by Curator (IC)
Experimental
Inferred from Mutant Phenotype (IMP)
Inferred from Direct Assay (IDA)
Inferred from Physical Interaction (IPI)
Inferred from Genetic Interaction (IGI)
Inferred from Expression Pattern (IEP)
Author statement
Non-traceable Author Statement (NAS)
Traceable Author Statement (TAS)
